# The Vagaries of the Drug Market

**Published:** 1908-03-14

**Authors:** 


### THE VAGARIES OF THE DRUG MARKET.

Several recent events in the drug market show very clearly that if economy is to be studied the market must be watched very closely by buyers. Fluctuations in value caused by the ordinary conditions of supply and demand are, as a rule, of such small dimensions as to have but little effect one way or the other on the drug bill of a practitioner whose requirements are comparatively light, but in the case of hospitals may make a substantial difference in the annual account. It is not, however, with price changes of an ordinary character that this note is concerned, but rather with one or two remarkable fluctuations, the causes of which are not of the usual nature. The experienced buyer was prepared for the recent heavy reduction in the price of santonin, although he was possibly surprised that the value was cut in half. On the other hand, the buyer who increased his stock on the day before the drop was announced, as more than one did, had reason for disappointment. Some years ago a syndicate obtained from the Russian Government the monopoly of the raw material, which grows wild in Russian Turkestan; at that time the price of the manufactured article was 6s. per lb., but as a result of the monopoly it steadily advanced to 40s. Before the intervention of the syndicate the nomad tribes who gathered the plant were free to sell it to whom they chose, but under the new conditions they were obliged to sell it to the syndicate, which fixed the purchasing price. In course of time these tribes became discontented and appealed to the governor of the province, who, it seems, decided that the monopoly in worm seed was against public policy and sanctioned the free sale of the seed. The product thus got into the hands of competing manufacturers; hence the two drops in the price of santonin which have recently occurred. The probability is that the competition will continue, and a further reduction should not come as a surprise, although the present price may be considered low. Not long ago the price of lithia salts was, without warning, reduced 50 per cent., this being probably the result of a tactical move on the part of established manufacturers who are desirous of placing an effective check on threatened competition. As makers are not anxious to book orders at the existing price, and other circumstances being taken into consideration, buyers would be well advised to take in sufficient stock to satisfy their requirements for some time to come, for the price may advance to its old position just as suddenly as it was reduced.

Owing to the partial failure of the poppy crop in Turkey the price of morphine advanced week by week until, towards the end of October the record figure of nearly 10s. per oz. was quoted for the hydrochloride. Similarly the price of codeine advanced to 16s. per oz. As holders of opium in the producing districts showed no indication of weakness there seemed every likelihood that the value of the alkaloids would remain firm, or even advance still further. Buyers who knew the opium market intimately, however, had noted that although the price of Turkey opium—the kind almost invariably used by manufacturers—was practically prohibitive, there was a fair supply of Persian opium in hand, and anticipated that makers of the alkaloids would fall back on this variety of raw material. This, in fact, happened, and as the Persian drug worked out at a lower cost than the Turkish, the morphine contents being compared, makers were able to reduce their prices for the alkaloids. Several times quotations have declined, the last reduction bringing the price of morphine hydrochloride down to 7s. 5d. per oz., and of codeine to 11s. 6d. On some hands it is suggested that makers have been influenced by the advent of a competing manufacturer, but this has not been confirmed. The possibility is that the tendency will continue to be in a downward rather than an upward direction, although at the time of writing the position of opium is somewhat firmer.

One of the most interesting recent events of the drug market has been the arrival of synthetic camphor of British manufacture. Synthetic camphor has been talked of for some time, but the difficulty hitherto has been to get any definite offers of supplies; the article is now freely offered at a price below that quoted by English refiners for the natural product. Although, until recently, it was impossible to obtain much information concerning synthetic camphor, the knowledge that it was likely to be produced had a very noticeable influence on the market for the Japanese product, the price of which declined so rapidly that it at last became possible for English refiners to sell at 2s. 10d. per lb. a product which a few months earlier was quoted at 4s. 9d. The general opinion is that the tendency in the future will be to lower prices.

The introduction into commerce of synthetic camphor raises an important question. In appearance the new product is identical with natural camphor, and they both answer the same chemical tests. There is this difference, however : while natural camphor is dextrorotatory the artificial variety has no influence on polarised light, and by this means the two products can be distinguished. The British Pharmacopœia describes camphor as "a white crystalline substance obtained from *Cinnamomium camphora*," and it is therefore evident that when B.P. camphor is prescribed synthetic camphor cannot be dispensed. It may be suggested to the compilers of the Pharmacopœia that in the next edition the monograph should be so altered as to admit of the use in medicine of the new substance. The demand for medicinal requirements is, however, small compared with the demand for industrial purposes, and the market price is not largely influenced by the amount of camphor consumed in remedial agents.

A noteworthy event in the drug market was the enormous offering of cinchona bark at a recent sale in Amsterdam. The bark that came under the hammer represented a total of considerably over a million kilogrammes, which constitutes a record. The equivalent contents of quinine sulphate in this bark was over two million three hundred thousand ounces. The bulk of the offerings found buyers, and in due course will pass into the hands of consumers in the form of quinine. These figures seem to disprove the assertion that quinine is going out of fashion owing to the advent of synthetic febrifuges.

